# Influence of Pumping Regime on Temperature Resolution in Nanothermometry

**DOI:** 10.3390/nano11071782

**Published:** 2021-07-09

**Authors:** Jonas Thiem, Axel Ruehl, Detlev Ristau

**Affiliations:** 1 Institute of Quantum Optics, Leibniz University Hannover, Welfengarten 1, D-30167 Hannover, Germany; thiem@stud.uni-hannover.de (J.T.); d.ristau@lzh.de (D.R.); 2 QUEST-Leibniz-Research School, Institute of Quantum Optics, Leibniz University Hannover, Welfengarten 1, D-30167 Hannover, Germany; 3 Cluster of Excellence PhoenixD, D-30167 Hannover, Germany

**Keywords:** laser rate equations, fluorescence lifetime, energy levels, nanothermometry

## Abstract

In recent years, optical nanothermometers have seen huge improvements in terms of precision as well as versatility, and several research efforts have been directed at adapting novel active materials or further optimizing the temperature sensitivity. The signal-to-noise ratio of the emission lines is commonly seen as the only limitation regarding high precision measurements. The role of re-absorption caused by a population of lower energy levels, however, has so far been neglected as a potential bottleneck for both high resolution and material selection. In this work, we conduct a study of the time dependent evolution of population densities in different luminescence nanothermometer classes under the commonly used pulsed excitation scheme. It is shown that the population of lower energy levels varies when the pump source fluctuates in terms of power and pulse duration. This leads to a significant degradation in temperature resolution, with limiting values of 0.5 K for common systems. Our study on the error margin indicates that either short pulsed or continuous excitation should be preferred for high precision measurements. Additionally, we derive conversion factors, enabling the re-calibration of currently available intensity ratio measurements to the steady state regime, thus facilitating the transition from pulse regimes to continuous excitation.

## 1. Introduction

Luminescence based nanothermometry has established itself as a versatile approach to measuring temperature with high resolution in both thermal and spatial dimensions. The selling points of this technology are easily identified, as it is a non-contact measurement and it can be used for voluminous measurements in doped media [1] and on surfaces [2]. Moreover, it is robust against many external influences, such as electric fields [1]. The fundamental quantity to measure in nanothermometry is the luminescence intensity ratio (FIR) of two emission lines. Temperature dependence of the FIR is ensured by choosing emission lines originating from thermally coupled energy levels, separated by ΔE. This leads to a Boltzmann dependency of the FIR given by

(1) $$\mathrm{FIR}\propto \frac{N_{1}}{N_{2}}\approx \exp\left(\frac{\Delta E}{k_{B}T}\right),$$

with N${}_{1}$ and N${}_{2}$ being the population densities of the two upper energy levels, k${}_{B}$ the Boltzmann constant, and T the temperature. The relative sensitivity

(2) $$S=\frac{1}{{\mathrm{FIR}}_{0}}\frac{d\mathrm{FIR}}{dT}$$

is then determined by the FIR at room temperature FIR${}_{0}$, and by the rate at which the FIR changes with temperature. The temperature resolution ΔT can be derived from Equation (2) to be

(3) $$\Delta T=\frac{1}{{\mathrm{FIR}}_{0}}\frac{1}{S}\Delta \mathrm{FIR}.$$

**Figure 1:**
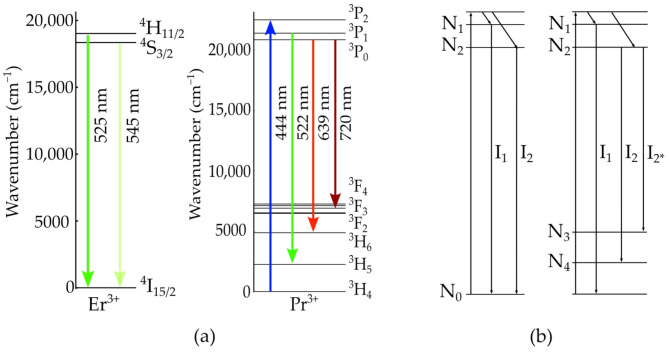
Energy levels of trivalent ions Er${}^{3+}$ and Pr${}^{3+}$ ions used in nanothermometry [6,7] with the optical transitions for excitation and emission in (**a**) and the corresponding 3-level and 4-level laser schemes in (**b**).

Based on this set of equations, material selection for nanothermometry seems to be straight forward, as the only criterion for inclusion should be a correctly chosen relation of Δ E/k${}_{B}$T [3]. Although this is sufficient for most practical applications, further effects need to be considered when aiming at an optimization of the resolution and a reduction of the setup’s complexity. For example, non-radiative relaxations may impose measurement errors if the transition rate is comparable to the thermal relaxation rate [4]. Absorption of the luminescence in the host material can cause errors exceeding 10 K [5] and needs to be considered, especially if the absorption strength is also temperature dependent. However, the pumping regime is mostly overlooked as a potential source of temperature errors although it strongly affects the population densities of the lower energy levels during the measurement. The influence of population densities can be illustrated by comparing the energy level configuration of two commonly used active ions, Erbium (Er${}^{3+}$) [6] and Praseodymium (Pr${}^{3+}$) [7], as depicted in Figure 1a. The combinations of transitions used in Er${}^{3+}$ nanothermometry are ${}^{4}$H${}_{11/2}\to^{4}$I${}_{15/2}$ centered around 525 nm with ${}^{4}$S${}_{3/2}\to^{4}$I${}_{15/2}$ centered around 545 nm, therefore including only one lower energy level. In contrast, using Pr${}^{3+}$ for nanothermometry enables different transitions, for example, ${}^{3}$P${}_{1}\to^{3}$H${}_{5}$ at 522 nm and ${}^{3}$P${}_{0}\to^{3}$H${}_{6}$ at 639 nm with varying lower energy levels. The same distinction applies to other active ions, such as Nd${}^{3+}$[3] or Ho${}^{3+}$ [8], respectively. The schematic 3- and 4-level laser diagrams provide a more general view and are shown in Figure 1b. One benefit of using varying lower energy levels is the spectral separation of the emission lines independent of the energy gap of the upper levels, therefore adding flexibility to measurements.

Currently, the sensitivity of nanothermometers is characterized either under pulsed excitation [7,8] or continuous excitation [1,3]. This raises the question of whether calibration data acquired with either of these systems are interchangeable, even for the same active ion. Re-absorption of emission could prevent this transfer because these population densities differ between instantaneous and steady state conditions. Re-absorption is a well-known phenomenon resulting, for example, in the reduced efficiency of Pr:YLF lasers emitting in the orange spectral range [9]. Fitting the thermal evolution of the FIR in Pr${}^{3+}$:YF${}_{3}$ for the same upper levels, but different lower laser levels, may yield different ΔE values [10], which could also be attributed to re-absorption differences. Consequently, a population of lower energy levels has to be considered as the origin of FIR deviations, even in the absence of temperature variations, therefore causing measurement errors. We addressed this issue by utilizing time dependent laser rate equations. Since the two main factors determining the population density in luminescence applications are the pumping rate and the emission lifetime, we derived the corresponding relationships for a population of lower energy levels. As a result of this, different calibration regimes for nanothermometry can be defined depending on the pump duration and luminescence lifetime. The pumping rate fluctuates through variations of either energy, wavelength or duration, and the corresponding error caused by re-absorption is presented for an exemplary Pr${}^{3+}$ based nanothermometer system. The main finding of our work is that either short pulses or continuous excitation should be preferred, as both help to improve temperature resolution. For practical applications, distinctions between the two regimes may be necessary depending on the desired sampling rate or complexity of the setup. Therefore, we developed a formalism to unify pumping schemes and to maintain baseline measurements.

## 2. Population Densities

**Figure 2:**
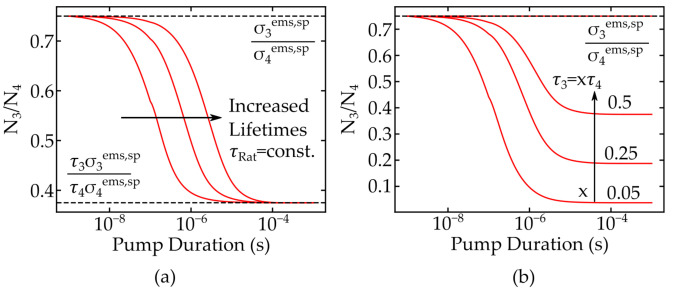
Relative relations of the population densities N${}_{3}$ and N${}_{4}$ of lower laser levels, as defined in Figure 1, depending on the pump duration. Depicted in (**a**) are relations for a constant ratio $\tau_{\mathrm{Rat}}$ of the emission lifetimes and in (**b**) for different ratios.

We used laser rate equations [11], as given in Appendix A, to calculate the population density of the emission levels N${}_{3}$ and N${}_{4}$ of a 4–level scheme as shown in Figure 1. Figure 2 illustrates the relations of these populations depending on the relative strength of the spontaneous emissions, which is determined by their emission cross sections $\sigma_{\mathrm{ems},2}$ and $\sigma_{\mathrm{ems},2*}$, and the lifetimes of the levels $\tau_{3}$ and $\tau_{4}$, respectively.

The material parameters were taken according to a Pr:LiLuF crystal [12] and the simulations were performed with a constant pump power of 1 W. The amplitude and temporal evolution of these changes is determined by the material parameters, as seen in Figure 2. The onset of the different stable regimes is determined by the absolute values of the lifetimes $\tau_{3}$ and $\tau_{4}$ and, for example, increasing the lifetimes delays the achievement of the steady state operation. This result is shown for a constant ratio $\tau_{\mathrm{Rat}}=\tau_{3}/\tau_{4}$ in Figure 2a. The difference of the population ratio depends on the ratio between the two lifetimes, as depicted in Figure 2b.

**Figure 3:**
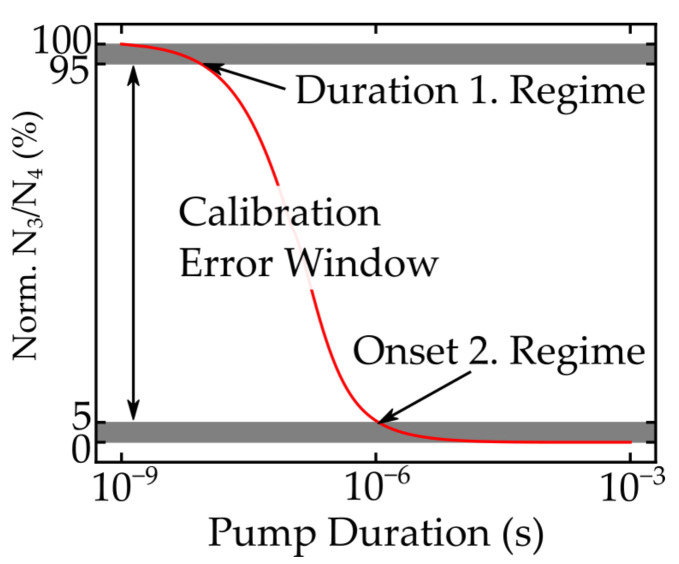
Exemplary depiction of the different calibration regimes caused by changes in energy level populations.

These relations can be used to identify application areas for nanothermometry. Figure 3 highlights that two stable regimes of the temporal evolution of N${}_{3}$/N${}_{4}$ exist, depending on the pump duration. For short excitation times, the population relation is defined only by their respective emission cross sections. The influence of the lifetimes $\tau_{3}$ and $\tau_{4}$ grows for longer pump duration. Once the steady state regimes of all involved energy levels are reached, the second stable regime commences. Generally, these results apply to all materials where the luminescence occurs at different, lower energy levels. For materials such as Er${}^{3+}$, however, this effect vanishes as the lifetime and cross sections of lower levels are identical and both regimes are the same. The population of lower energy levels and the FIR error are connected through the reabsorption of luminescence in order to transfer these results to a temperature measurement. The corresponding cross section $\sigma_{\mathrm{abs}}$ was calculated using the McCumber relation [13] from $\sigma_{\mathrm{ems}}$, Z${}_{1}$ and Z${}_{2}$ as parameters for the degeneracy of the Stark levels in the two involved energy levels 1 and 2, and the energy distance E${}_{0}$ between the lowest energy levels of both manifolds 1 and 2 [14]. The relation is defined as follows:

(4) $$\sigma_{\mathrm{ems}}\left(\nu \right)≃\sigma_{\mathrm{abs}}\frac{Z_{1}}{Z_{2}}\exp\left[\left(\frac{E_{0}-h\nu }{k_{B}T}\right)\right],$$

and material parameters were chosen for a Pr${}^{3+}$ nanothermometer. This formalism gives a proportionality between the absorption and emission cross section, with an additional factor of Stark level splitting and population [15]. It is therefore used to gain an estimation of the remaining luminescence P(z) after absorption via the Lambert–Beers Law. Applying the approximation for a short interaction length z yields

(5) $$P\left(z\right)=P_{0}\exp\left(-N\sigma_{\mathrm{abs}}z\right)\approx P_{0}\left(1-N\sigma_{\mathrm{abs}}z\right).$$

Based on these relations and Equation (1), a factor C to convert the instantaneous FIR${}^{\mathrm{inst}}$ to the steady state FIR${}^{t\infty }$ is defined:

(6) $$\begin{array}{cc} {\mathrm{FIR}}^{t\infty } & ={\mathrm{FIR}}^{\mathrm{inst}}C \end{array}$$

(7) $$\begin{array}{cc} & ={\mathrm{FIR}}^{\mathrm{inst}}\frac{1-\sigma_{\mathrm{abs},1}N_{1}z}{1-\sigma_{\mathrm{abs},2}N_{2}z}. \end{array}$$

## 3. Influence of Pump Fluctuations in Pulsed Regime

Pulsed excitation sources operating in the nanosecond regime are frequently used in nanothermometry [7,8], and typical pump sources, such as OPOs or q-switched lasers, are prone to power and energy fluctuations, respectively. As the value of $\sigma_{\mathrm{abs}}$ is wavelength dependent, fluctuations of the emission wavelength can also be treated as energy fluctuations, as can be seen from Equation (5). Furthermore, the pulse duration FWHM${}_{\mathrm{EXC}}$, corresponding to a Gaussian shaped pulse, may also fluctuate, resulting in another source of measurement errors. The temporal evolution of luminescence emission is simulated to characterize these effects and to gain the amplitude of ΔFIR.

**Figure 4:**
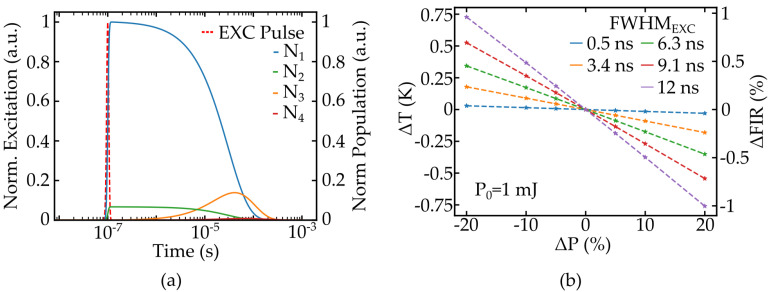
Temporal evolution of population density calculated for approximated Pr${}^{3+}$ (**a**) and the resulting temperature error ΔT for FIR measurements depending on pump energy fluctuations ΔP in (**b**).

**Figure 5:**
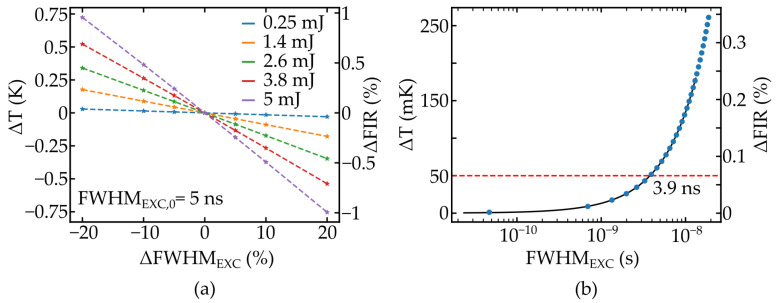
Temporal fluctuations of the excitation pulse width FHWM${}_{\mathrm{EXC}}$ and the resulting temperature error for the FIR measurement (**a**) and the estimation of the achievable resolution depending on pulse duration (**b**).

The corresponding temperature error ΔT, caused by the fluctuations, can be accessed by using the specifications of Pr:YLF nanothermometers with FIR${}_{0}\approx$ 1.2 and S ≈ 1.1 %/K [7] through Equation (3). The lifetimes of energy levels for a given dopand also depends on the host material. Unfortunately, the lifetimes of Pr:YLF crystals were not available, so the lifetimes of the ${}^{3}$F${}_{3}$ or ${}^{3}$F${}_{4}$ levels in Pr:LaCl${}_{3}$, reported to be 58 μs [16], were used for the calculations. However, the proposed formalism is still valid for different lifetimes, and the calculations can easily be adapted after measuring the transition rates inside the desired material. The results are shown in Figure 4 and Figure 5.

As seen in Figure 4a, the populations of the thermally coupled emission levels N${}_{1}$ and N${}_{2}$ reach their respective maximums closely following the excitation pulse, whereas the populations of the lower levels N${}_{3}$ and N${}_{4}$ are shifted due to the lifetime of the upper levels, as expected. The temperature error caused by the different absorptions of luminescence emission over time is given in Figure 4b and Figure 5a, depending on energy variation ΔP and pulse duration fluctuations ΔFWHM${}_{\mathrm{EXC}}$, respectively. The amplitude of the error is similar and, for both fluctuations, ranges of 20% are identified to cause a temperature error of approximately 0.75 K. This error ΔT does not directly correlate to the actual resolution achievable in a measurement as additional noise sources need to be factored in. Therefore, this value should rather be considered as a minimum value. It becomes apparent that in order to perform high precision temperature measurements, pump rate fluctuations need to be minimized by using stabilized short pulse pump lasers, although even stabilized OPO systems exhibit ±6% energy fluctuations [17].

The dependency of ΔT on pulse duration and a constant energy fluctuation of 10% are shown in Figure 5b; a pump pulse duration of below one nanosecond should be preferred.

**Figure 6:**
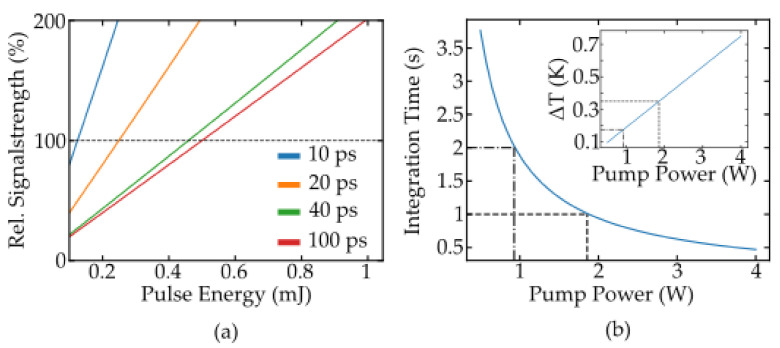
Requirements for pulse energy (**a**) and pumping power (**b**) to maintain the signal strength of the 3.9 ns excitation marked in Figure 5 and the temperature deviation caused by laser absorption (inset b).

Apart from pump noise, the overall error budget is also influenced by the strength of the emitted luminescence. Therefore, switching the pump regime may result in the need to also adjust the pump energy or power, respectively. Simulations were performed for both short pulsed lasers (>>1 ns) and continuously emitting diodes to obtain these values and to maintain the signal strength of the assumed 3.9 ns pulse, marked in Figure 5b. Therefore, the most relevant in these simulations is the strength of luminescence emission, which again is proportional to the population densities. For the short pulsed laser systems, three pulse durations of 20 ps, 50 ps and 100 ps were chosen and the total emission is compared relative to the aforementioned nanosecond pulse. For the continuous case, the integration time was included as a parameter to identify the required combinations of integration time and pump power for the desired signal strength. The results are shown in Figure 6 and highlight the two optimum pump regimes for nanothermometry: short pulse and continuous wave excitation. The pulse energy needed for an excitation duration of 100 ps is approximately 0.5 mJ, and this pump regime can be used for high precision measurements even without the exact knowledge of material parameters like the emission lifetimes. However, especially for cost effective measurements, this approach can be undesired as it requires more complex laser sources. A continuous excitation is hence favorable. On the other hand, the integration time necessary to achieve the same signal strength with a simplified setup using a continuous emitting laser diode lies in the range of 1–2 s and is thus not applicable for high sampling rates. The increased heat input by the pump laser is another potential drawback of continuous excitation. The steady state temperature deviation caused by 1–2 W incident pump power can be estimated with approximately 0.15 K–0.35 K, as depicted in the inset of Figure 6. The quantum defect of the Pr:YLF emission was considered to be the main heat source, and doped SiO${}_{2}$ was assumed to be the host material in this calculation. The formalism used for this estimation can be found in [18]. Additional absorption, for example, by the host material, may further increase ΔT but the measurement error caused by this can be easily calibrated as shown in [1].

## 4. Calibration for Different Pump Regimes

Both of the presented pumping regimes offer advantages and disadvantages, and their usage should be carefully chosen depending on the desired application. Since most baseline measurements that are currently used are conducted with either pulsed or continuous excitation, the formalism presented in Equation (6) can be used to transfer baseline measurements. This procedure is described for a switch from pulsed to continuous excitation in the following.

**Figure 7:**
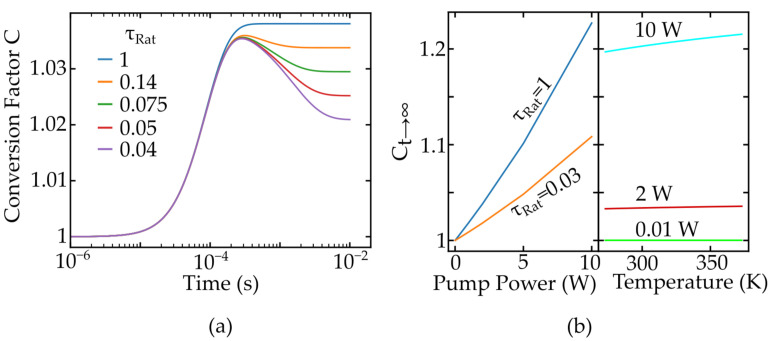
Conversion factor C depending on time and the ratio of lifetimes $\tau_{\mathrm{Rat}}$ of the lower laser levels in (**a**), and shown in (**b**) is the value of C for long times depending on the temperature and the pump power.

The first step is to measure the lifetimes of the lower levels, giving access to the ratio $\tau_{\mathrm{Rat}}$ of lifetimes of the lower energy levels. In Figure 7a, the conversion factor is again given for different values of $\tau_{\mathrm{Rat}}$, with again one value fixed to 58 μs. The temporal evolution is explained by the different onsets of the steady state regime combined with varying absorption strengths of the emission lines causing local extreme values. Once the material is identified, the main parameters that are varied during the measurements are the pump power and the temperature of the surrounding media. The pump power is used to improve the signal-to-noise ratio, and the temperature needs to be included because reabsorption also depends on temperature via the Stark level population, as seen in Equation (4). The dependency of re-absorption on the temperature is especially important for the formalism, as it shifts the assumed single exponential Boltzmann dependency of the FIR on temperature given in Equation (1).

**Figure 8:**
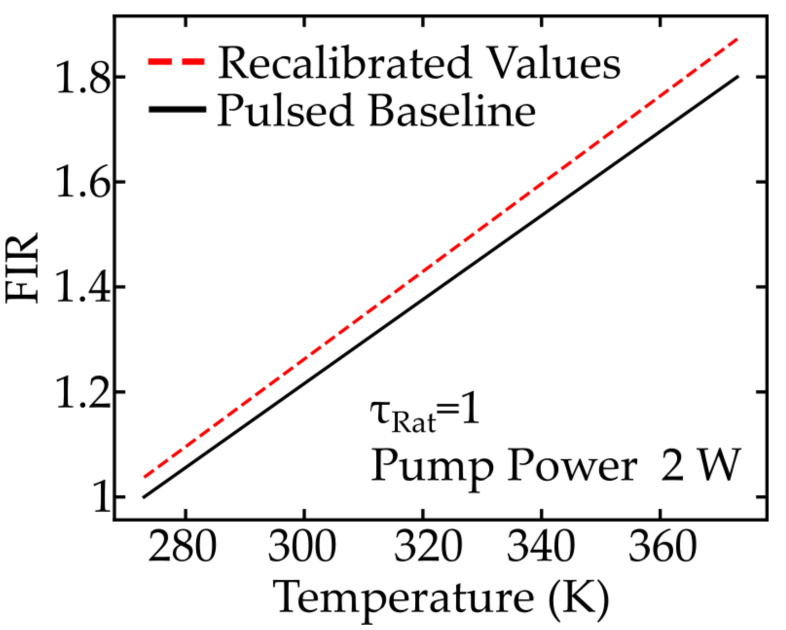
Typical FIR evolution for Pr:YLF nanothermometers [7] measured with pulsed excitation in black and the re-calibrated version determined by applying the correction factor C.

This shift factor is applied to an approximated FIR evolution for the Pr:YLF nanothermometers [7] and the results are shown in Figure 8. As depicted, the correction of FIR therefore helps to prevent a systematic overestimation of the measured temperature in cw-measurements.

## 5. Conclusions and Outlook

We developed a formalism to incorporate the influence of the pumping regime into luminescence nanothermometry measurements. We showed that commonly used nanosecond pump lasers, such as OPOs or Q-switch lasers, impose a limit on temperature resolution as shown by the numerical study of measurement errors when neglecting re-absorption. Both short pulsed (<<1 ns) and continuous excitation schemes are able to bypass this bottleneck. Viable reasons for the application of either pumping regime can be found in the desired sampling rate or the setup complexity. For example, the measurement of rapidly changing temperatures should be conducted with a high sampling rate and therefore pulsed excitation. For the monitoring of more stable temperatures, for example, in industrial applications, a combination of solids doped with nanocrystals and a continuous emitting laser diode [1] may be preferred due to cost effectiveness. A calibration formalism was presented to maintain already existing baseline measurements, enabling a change in the pumping regime without the need for additional calibration measurements.

## Data Availability

The data presented in this study may be obtained from the authors upon reasonable request.

## Appendix A

The numerical calculations were performed using Python. A system of rate equations was used to model the presented data numerically [11,19]. For an efficient numerical implementation, a Finite-Difference Scheme was used. Besides this, no further approximations have been involved. The fundamental equations are those for population densities, here exemplary N${}_{2}$ and N${}_{1}$, and the laser power P inside the system. The time dependent variations of two population densities is given by

(A1) $$\begin{array}{cc} \frac{dN_{2}}{dt} & =-\frac{N_{2}}{\tau }+\left(\frac{\sigma_{\mathrm{abs}}\left(\lambda \right)P\left(z,\lambda \right)}{h\nu A}N_{1}-\frac{\sigma_{\mathrm{ems}}\left(\lambda \right)P\left(z,\lambda \right)}{h\nu A}N_{2}\right), \end{array}$$

(A2) $$\begin{array}{cc} \frac{dN_{1}}{dt} & =-\left(\frac{\sigma_{\mathrm{abs}}\left(\lambda \right)P\left(z,\lambda \right)}{h\nu A}N_{1}+\frac{\sigma_{\mathrm{ems}}\left(\lambda \right)P\left(z,\lambda \right)}{h\nu A}N_{2}\right), \end{array}$$

(A3) $$\begin{array}{cc} N_{\mathrm{total}} & =N_{1}+N_{2}. \end{array}$$

The parameters used in this calculations are the fluorescence lifetime τ of the energy level $N_{2}$, the cross sections $\sigma_{\mathrm{abs}}$ and $\sigma_{\mathrm{ems}}=\sigma_{\mathrm{stim}}+\sigma_{\mathrm{sp}}$ for absorption, spontaneous, and stimulated emission, respectively, *A* is the pumped area. Additional energy levels are added following the same scheme by a combination of terms for increase and decrease of populations. The laser power is determined by

(A4) $$\frac{dP}{dz}=\sigma_{\mathrm{stim}}\left(\lambda \right)N_{2}P\left(z,\lambda \right)-\sigma_{\mathrm{abs}}\left(\lambda \right)N_{1}P\left(z,\lambda \right)+\frac{N_{2}hc}{\tau \lambda }\sigma_{\mathrm{sp}}\left(\lambda \right)\beta A.$$

where β is a variable that gives the solid angle of the considered spontaneous emission.

**Table A1:** Parameters used for the simulation of the population densities in Praseodymium doped nanothermometers all values are according to the cited literature [12,16]

Parameter	Value
$\sigma_{\mathrm{abs}}$ (444 nm)	5.8 × 10${}^{-24}$ m${}^{2}$
$\sigma_{\mathrm{ems}}$ (522 nm)	1.2 × 10${}^{-24}$ m${}^{2}$
$\sigma_{\mathrm{ems}}$ (639 nm)	6.9 × 10${}^{-24}$ m${}^{2}$
$\sigma_{\mathrm{ems}}$ (720 nm)	3.1 × 10${}^{-24}$ m${}^{2}$
τ(${}^{3}$F${}_{4}$)	58 μs
Pr:YLF Doping	0.65 at%
